# Socio-demographic and therapeutic profile of people living with thalassemia in Brazil between 2012 and 2022: A cross-sectional study

**DOI:** 10.1016/j.htct.2026.106461

**Published:** 2026-05-02

**Authors:** Janaína Rosenburg Gioseffi, Fernanda Cristina dos Santos Simão, Nina Melo, Eduardo Maercio Fróes, Talita Garrido Araújo, Fábio Augusto Fedozzi, Catherine Moura Fonseca Pinto, Sandra Loggetto

**Affiliations:** a Associação Brasileira de Talassemia – Abrasta. São Paulo, SP, Brazil; b Banco de Sangue de São Paulo – Grupo GSH. São Paulo, SP, Brazil

**Keywords:** Beta thalassemia, Blood transfusion, Rare disease

## Abstract

**Objective:**

To outline the sociodemographic and therapeutic profile of individuals with thalassemia in Brazil between 2012 and 2022, by analyzing data from the Ministry of Health on outpatient care, hospitalization, and mortality records.

**Methods:**

This descriptive, cross-sectional study utilized secondary data from the Outpatient, Hospital, and Mortality Information Systems of the Ministry of Health on patients diagnosed with thalassemia (ICD-10 D56). Analyzed variables included demographics (sex, age, race/ethnicity), geographic location (residence and treatment site), and clinical outcomes (procedures, hospitalizations, and deaths).

**Results:**

In 2022, an estimated 662 patients received outpatient treatment in the Brazilian Unified Health System (SUS), with a predominance of young adults, women, and individuals self-identified as White, who were mostly concentrated in the southeastern and southern regions of the country. Red blood cell transfusion was the most frequently performed outpatient procedure during the period. There were 1278 hospitalizations, mainly of children aged 0–9 years. A total of 153 deaths were recorded, primarily among people aged 50 years or older. The findings characterize the patient profile, identify significant travel challenges to receive treatment, and suggest potential underreporting within reference institutions.

**Conclusion:**

It is essential to improve data recording in public databases to support specific health policies and ensure comprehensive, equitable, and continuous treatment for people living with thalassemia in Brazil.

## Introduction

Thalassemia is a hemoglobinopathy, meaning a genetic condition characterized by the absence or significant reduction in the production of one or more globin chains (alpha or beta) that make up hemoglobin [1,2]. In the most severe form of the disease, the red blood cells (RBCs) produced are more fragile and have a shorter lifespan than normal, resulting in severe anemia [3].

Alpha thalassemia, in which the primary defect is in the production of alpha globin chains, presents as four clinical forms: silent carrier (without clinical manifestations), alpha thalassemia trait (mild anemia), hemoglobin H disease (moderate to severe anemia), and hydrops fetalis (extremely severe and incompatible with life) [4]. Caused by a defect in beta-globin chain synthesis, beta thalassemia presents as three distinct clinical phenotypes: beta thalassemia minor or thalassemic trait (generally asymptomatic), thalassemia intermedia (mild to severe anemia that may require RBC transfusions), and thalassemia major (severe anemia requiring continuous RBC transfusions every 2–4 weeks from the first months of life) [3,4]. The trait forms of thalassemia occur when only one parent is a carrier of the gene for the disease, while the more severe forms occur when both parents are carriers of the alpha or beta thalassemia gene [3,4].

Beta thalassemia is common in the Mediterranean region, the Middle East, and Asia. Alpha thalassemia is more common in Southeast Asia. Migration resulting from globalization has introduced hemoglobinopathies to areas where they were not originally endemic [2]; in Brazil, this occurred mainly through the arrival of Greeks and Italians to the country, with the promise of a better life, in the mid-19th century [4].

Currently, about 5% of the world population carries a gene for some abnormal hemoglobin [2]. In Brazil, it is estimated that 1.1% of the population may have genetic alterations for thalassemia [1]. The Brazilian Thalassemia Association (Abrasta) estimates that about a thousand people in the country are living with severe forms of thalassemia. Among registered individuals with thalassemia, 283 have beta thalassemia major and 222 have beta thalassemia intermedia. These data highlight the importance of proper follow-up and treatment for affected patients [3].

Since early diagnosis is necessary for proper treatment [5], thalassemia is detected via newborn screening at birth and confirmed using hemoglobin electrophoresis. The specific genetic mutation test is rarely used in Brazil due to limited access to this exam [4].

Treatment and follow-up for individuals living with thalassemias are essential to prevent disease-related complications, such as growth retardation, splenomegaly, hepatomegaly, weakened musculature, leg ulcers, extramedullary hematopoiesis, bone deformities of the legs, and craniofacial changes. To prevent these complications, people with beta thalassemia major undergo regular RBC transfusions throughout their lives to maintain adequate hemoglobin levels and minimize symptoms associated with anemia [3,5]. Treatment for intermediate beta or alpha thalassemias may include sporadic or frequent RBC transfusions, depending on each patient’s clinical progression [3,5]. Chelation therapy, to manage the iron overload resulting from frequent transfusions, is also an integral part of the treatment plan, in addition to a multidisciplinary approach involving hematologists, palliative care specialists, and other healthcare professionals to provide comprehensive and personalized care [3,5].

Given that thalassemia is a rare disease with limited epidemiological data in Brazil, this study aimed to outline the sociodemographic profile of affected individuals between 2012 and 2022. Additionally, it analyzes outpatient and hospital production, focusing on primary procedures, and assesses mortality rates associated with the condition during the same period.

## Methods

A cross-sectional descriptive study was conducted using secondary data from the period from 2012 to 2022 encompassing all types of thalassemia (diagnostic codes D56.0 through D56.9) according to the International Statistical Classification of Diseases and Related Health Problems (ICD-10).

Based on this, categories D56.0 and D56.3 of the ICD-10, which refer to the alpha and beta thalassemia traits, respectively and do not require outpatient treatment, were excluded. Data were retrieved from the Ministry of Health's public databases: the Outpatient Information System (OIS) for ambulatory procedures, the Hospital Information System (HIS) for admissions, and the Mortality Information System (MIS) for mortality data.

The most up-to-date consolidated data in the public databases are related to 2022. Therefore, a retrospective analysis of a decade from 2012 to 2022 was chosen. The datasets were subjected to a cleaning and organization process using Microsoft Excel® to select variables for this study. These variables included sex, age, race/ethnicity, city of residence, city of treatment, state where the procedure/hospitalization took place, cost of procedure/hospitalization, type of procedure performed, number of procedures, year of diagnosis, year the procedure was performed, year of hospitalization, and year of death.

Age was stratified into the following groups: 0–9, 10–19, 20–29, 30–39, 40–49, and ≥ 50 years. The three most prevalent procedures were specifically identified and all other procedures were grouped as 'Others' for the purpose of analysis.

The number of users with thalassemia registered in SUS was estimated by cross-referencing information such as the municipality of residence, sex, ICD-10 category, and the healthcare institution responsible for treatment. To focus on relevant procedures, all types of procedures were selected, ranging from clinical and imaging examinations to blood transfusions and patient follow-up procedures.

It is important to note that the data collected refers exclusively to outpatient and hospital services linked to thalassemia as provided by SUS, and does not include information from the supplementary health network. The public databases were accessed on the data systematization website of the Ministry of Health (available at: https://datasus.saude.gov.br/transferencia-de-arquivos).

In compliance with Brazilian Law No. 12,527/2011 and CNS Resolution 510/2016, this study did not require Research Ethics Committee approval as it used publicly accessible secondary data that contains no personally identifiable information.

## Results

### Outpatient information system (OIS)

Within the scope of the OIS, an estimated 662 patients underwent outpatient treatment in 2022, with São Paulo having the highest number at 244 estimated patients, followed by Rio Grande do Sul with 113. In contrast, Acre, Roraima, and Rio Grande do Norte did not report any patients in 2022 (Table 1).

**Table 1:** Estimated number of thalassemia patients in Brazil in 2022.

State	Estimated Patients	
	n	%
São Paulo	244	36.9
Rio Grande do Sul	113	17.1
Minas Gerais	107	16.3
Paraná	23	3.5
Distrito Federal	21	3.2
Mato Grosso	21	3.2
Santa Catarina	20	3.0
Maranhão	18	2.7
Piauí	18	2.7
Goiás	15	2.4
Rio de Janeiro	13	2.0
Espírito Santo	9	1.4
Pará	9	1.4
Bahia	6	0.9
Tocantins	6	0.9
Alagoas	4	0.6
Paraíba	3	0.5
Rondônia	3	0.5
Amapá	2	0.4
Ceará	2	0.4
Mato Grosso do Sul	2	0.4
Amazonas	1	0.2
Pernambuco	1	0.2
Sergipe	1	0.2
Acre	0	0.0
Roraima	0	0.0
Rio Grande do Norte	0	0.0
Total	662	100

Between 2012 and 2022, women accounted for 54.5% of the patients. The main age groups were 20–29 years (29%) and 30–39 years (24.9%), and most patients self-identified as White (86.5%). It is important to note that the majority of patients (64.3%) underwent procedures in cities other than where they resided (Table 2).

**Table 2:** Profile of outpatient procedures performed for thalassemia in Brazil between 2012 and 2022.

Variable	Outpatient Procedure
	**n (%)**
Race/Ethnicity	
White	115,817 (86.5)
Brown (*Pardo*)	8059 (6.0)
Black	3020 (2.3)
Asian	1450 (1.1)
Indigenous	217 (0.2)
Not declared	5330 (3.9)
Age Group (years)	
0–9	11,662 (8.7)
10–19	27,702 (20.7)
20–29	17,015 (29.2)
30–39	39,190 (24.9)
40–49	9426 (7.1)
≥50	12,612 (9.4)
Gender	
Male	61,020 (45.5)
Female	72,873 (54.5)
Type of procedure	
Red blood cell concentrate transfusion	53,124 (39.7)
Pre-transfusion Tests I*	39,619 (29.6)
Pre-transfusion Tests II⁎⁎	23,710 (17.7)
Others	17,440 (13.0)
Region of Brazil	
North	2068 (1.5)
Northeast	6852 (5.1)
Central-West	1610 (1.2)
South	32,143 (24.1)
Southeast	91,220 (68.1)
**Total**	**133,893**

⁎ Determination of ABO blood group and RH system D antigens, as well as the screening for irregular antibodies using the human antiglobulin method accompanied by an auto-control.

⁎⁎ Indirect antiglobulin technique for blood compatibility, also known as crossmatching.

A total of 133,893 procedures were performed, with RBC concentrate transfusions being the most frequent. Hospital das Clínicas at Unicamp in Campinas led with a number of nearly 50,000 procedures. In all, 233 facilities were identified as outpatient care locations. The Southeast was the Brazilian region with the highest number of procedures performed for thalassemia (68%), with São Paulo being the State with the largest volume of procedures (86,154). The southern region was ranked second with 24.1% of the outpatient production; Paraná accounted for 29,379 procedures (Table 2).

The year with the highest number of procedures was 2013, with 15,039, while the lowest was in 2020, possibly impacted by the Covid-19 pandemic, when 9713 procedures were recorded. The highest total cost, which occurred in 2022, exceeded two million reals, with an average cost of R$305.31 per procedure.

## Hospital information system (HIS)

In the HIS, a total of 1278 procedures were recorded during hospitalizations, with ‘treatment of hemolytic anemia’ being the most common. Women represented the majority of hospitalizations (50.2%), with the 0–9 years age group registering the highest number of cases (431 hospitalizations) (Table 3). About 40% of hospitalized patients lived in cities different to where they were hospitalized, with São Paulo being the State with the highest number of hospitalizations (532). Only 3% of the hospitalizations resulted in admission to an intensive care unit.

**Table 3:** Profile of hospital admissions for thalassemia in Brazil from 2012 to 2022.

Variable	Hospitalization
	**n (%)**
Race/Ethnicity	
White	545 (42.6)
Brown (*Pardo*)	424 (33.2)
Black	47 (3.7)
Asian	10 (0.8)
Indigenous	1 (0.1)
Not declared	251 (19.6)
Age Group (years)	
0–9	431 (33.8)
10–19	233 (18.2)
20–29	170 (13.3)
30–39	132 (10.3)
40–49	89 (7.0)
≥50	223 (17.4)
Gender	
Male	637 (49.8)
Female	641 (50.2)
Region of Brazil	
North	57 (4.7)
Northeast	234 (18.2)
Central-West	83 (6.4)
South	128 (10.0)
Southeast	776 (60.7)
**Total**	**1278**

The Santiago Children's Hospital in Natal, Rio Grande do Norte, recorded the highest number of procedures (n = 53) during hospitalizations. In Brazil, the year with the most hospitalizations was 2017 with 160 cases, while 2014 and 2015 each had 109 hospitalizations, and 2021 had 105 hospitalizations. The highest total cost occurred in 2014, exceeding 750,000 reals, with an average hospitalization cost of 2382.99 reals. There were 28 hospitalizations for allogeneic hematopoietic stem cell transplantations (HSCT) during the study period, with this procedure alone costing the public treasury more than two million reals. Hospital das Clínicas de Ribeirão Preto accounted for 23 hospitalizations for transplants.

## Mortality information system (MIS)

During the study period, Brazil recorded a total of 153 deaths related to thalassemia. Women (57.5%) and individuals aged 50 years or older (37%) accounted for the majority of fatal cases, while 67.3% of the deceased self-identified as White (Table 4). São Paulo had the highest number of deaths, totaling 56 cases. The years 2017 and 2020 had the highest numbers of deaths, with 19 cases each.

**Table 4:** Mortality profile of thalassemia in Brazil between 2012 and 2022.

Variable	Death
	**n (%)**
Race/Ethnicity	
White	103 (67.3)
Brown (*Pardo*)	34 (22.2)
Black	5 (3.3)
Not declared	11 (7.2)
Age group (years)	
0–9	10 (6.5)
10–19	10 (6.5)
20–29	10 (6.5)
30–39	21 (13.7)
40–49	13 (8.5)
≥50	89 (58.3)
Gender	
Male	65 (42.5)
Female	88 (57.5)
Region of Brazil	
North	6 (3.9)
Northeast	25 (16.4)
Central-West	10 (6.5)
South	22 (14.4)
Southeast	90 (58.8)
**Total**	**153**

## Discussion

Data collected between 1970 and 2019 from Italy, a country recognized for its high incidence of thalassemia, reported 7169 patients with thalassemia (5205 of whom had transfusion-dependent thalassemia [TDT] and 1964 with non-transfusion-dependent thalassemia [NTDT]). The majority of the patients were in the 36–50 age group and there were significantly more women than men. The survival cohort included 3777 patients; those with TDT had lower survival rates than NTDT patients (p-value <0.0001). Of the TDT group, the best survival rates were observed among women (71.8 years versus 68.8 years for men). A total of 523 deaths were recorded over the study period. Compared to the general population, patients with TDT had an increased risk of death, while the risk for NTDT was similar to that of the general population [6].

Analyses of outpatient production in the present study identified 662 patients in 2022. The results showed that the profile of people living with thalassemia in Brazil corresponds mostly to young adults aged between 20 and 29, followed by those aged 30–39, with the majority being women and originating from the southern and southeastern regions. São Paulo (Southeast) and Paraná (South) were the predominant states, accounting for 94.4% and 91.4% of all procedures within their respective regions. These factors demonstrate that most people living with thalassemia were born in the 1980s, 1990s and 2000s, when transfusional treatment, typically every two to four weeks, and iron chelation therapy were being established and becoming more widely available both in Brazil and worldwide [7, 8, 9]. The treatment protocol enabled a better quality of life and longer survival for people with TDT, preventing death as a consequence of anemia due to lack of treatment, since it is a disease incompatible with life [7, 8, 9].

The findings of the current study indicate that while the highest volume of outpatient procedures and estimated patient numbers are concentrated in states with significant Italian heritage, individuals with thalassemia are present across the country due to historical miscegenation and internal migration [1, 10, 11]. In several states across the North and Northeast, the majority of procedures were performed on individuals identified as ‘*Pardo*’ (Brown). This contrasts with other Brazilian regions, where the highest percentage of procedures occurred among the white population. The estimated number of patients for 2022 aligns closely with Abrasta’s projections. However, as these data only include individuals treated within SUS, they do not account for those receiving care through private health insurance [3].

Packed RBC transfusions were the most prevalent procedures, highlighting the importance of transfusional support in managing thalassemia. However, it is notable that this number was far below what was expected, considering that each patient on a transfusion protocol is estimated to undergo 12–24 transfusions annually, depending on their needs [8]. Some reference healthcare institutions for outpatient thalassemia treatment, such as Hemorio in Rio de Janeiro and Hemope in Pernambuco, do not have their data recorded in the OIS. This underreporting makes it difficult to estimate the number of patients, their treatments, and to fully understand the real situation of thalassemia in the country.

It is interesting to note the drop in the number of procedures in 2020, possibly due to the COVID-19 pandemic, which may have impacted access to health services and outpatient procedures for different conditions, including thalassemia [12].

It is also important to highlight the lack of reporting high-cost procedures involving medications such as iron chelators in the OIS. People with thalassemia use chelators to treat the buildup of iron in the body resulting from transfusions. Each unit of packed RBCs contains between 200 and 250 mg of iron, which is not metabolized by the body and accumulates in organs such as the liver, pancreas, and heart. If the excess iron is not eliminated from the body by iron chelation medications, the patient will develop secondary diseases such as liver cirrhosis, diabetes, and even heart failure [8,13]. Therefore, better articulation and integration of the data is important for unifying information on procedures for ICD-10 [11]. It is known that many patients have difficulty obtaining iron chelators through the public health system, often due to shortages or government bureaucracy [3]. By assessing availability of high-cost medications, it is possible to identify whether patients are truly receiving comprehensive treatment and to plan public policies to ensure the supply of this essential medication for the survival and quality of life of people with thalassemia, thereby preventing legal action.

Most thalassemia patients did not undergo procedures in the city where they reside, indicating a need to travel to major centers to access specialized health services [14]. There is also a notable predominance of women among the patients treated, which suggests a possible difference in access to health services between men and women, since it is known that women tend to be more concerned than men about regularly visiting the doctor and undergoing necessary exams and treatments to maintain their health [15].

Based on the HIS, the mean number of hospitalizations annually was 128 people, with a total of 1278 hospitalizations over the ten years of the study. In 2022, an estimated 19.3% of patients required hospitalization. However, specific triggers or procedures associated with these admissions could not be identified, as the majority were recorded under the generic ICD-10 classification 'treatment of patient with hemolytic anemia,' which lacks clinical detail regarding the circumstances of hospitalization. This demonstrates a lack of coordination among the available databases and raises doubts about the actual number of patients receiving outpatient and inpatient treatment.

Regarding specialized interventions, 28 hospitalizations for allogeneic HSCT were recorded among 26 patients. Allogeneic HSCT may be indicated for transfusion-dependent children and adolescents who do not have severe organ impairment (liver, heart) and have a 100% compatible donor, usually a sibling without the disease or with thalassemia minor [8,13].

Mortality data reveal a substantial disease burden, with 153 deaths recorded during the study period. This figure is notably high relative to the estimated 600 patients currently receiving treatment through the SUS, as identified by both this study and Abrasta data [3]. Unfortunately, the data did not identify the causes of death. It is worth remembering the secondary diseases result from transfusion treatment and iron overload. Death from heart failure secondary to iron accumulation remains a reality worldwide [7,8].

The majority of deaths occurred in individuals over 50 years of age (58%). This trend suggests that with optimized transfusion protocols, effective iron chelation, and multidisciplinary care, extended longevity is increasingly attainable. Consequently, the disease should not be viewed as a death sentence [8,13].

This study has limitations, as it relied on secondary data, leaving the authors dependent on the data entered by the teams responsible for treatment. Therefore, it does not fully represent the true number of people living with thalassemia and receiving transfusions via SUS.

The increased mortality among people with hemoglobinopathies, compared to the general population highlights the need for new therapies and individualized approaches to optimize treatment [6]. Luspatercept, approved in Brazil in 2021, represents an alternative adjunctive therapy for TDT and is intended for use in conjunction with regular transfusions [16,17]. Favorable results have already been published, despite the treatment remaining under clinical study with Brazilian participation [18]. In addition to allogeneic HSCT, curative strategies such as gene therapy are currently under development. These advancements may offer patients transfusion independence and an improved quality of life [19, 20, 21, 22], with the expectation of their future integration into the Brazilian healthcare system.

## Conclusion

People living with thalassemia in Brazil face invisibility due to the small number of diagnosed cases and the lack of better data in public reporting systems, which hinders a clear understanding of the disease. There is a need for commitment to improve data collection, especially regarding outpatient care, since these are chronic patients who are regularly followed in hematology departments. In the future, improved data might provide a solid foundation for developing health policies aimed at the prevention, diagnosis, and timely treatment of thalassemia in Brazil.

## Financial support

The study was supported institutionally by the Brazilian Association of Thalassemia (Abrasta)

## Data availability

The data that support the findings of this study are available from the corresponding author upon reasonable request.

## Conflicts of interest

The authors declare no conflicts of interest.
